# Meeting of the American Medical Association

**Published:** 1874-04

**Authors:** Wm. B. Atkinson

**Affiliations:** Permanent Secretary


### Editorial.

### Meeting of the American Medical Association.

The American Medical Association will hold its twenty-fifth annual session in Detroit, Michigan, on Tuesday, June 2d, 1874. The usual committees are expected to report upon the various subjects referred to them.

The following amendments to the Plan of Organization are to be acted upon:

By Dr. N. S. DAVIS, Illinois:

Strike out the second paragraph of Art. II. and insert the following:

"The delegates shall receive their appointment from permanently organized State Medical Societies, and such County and District Medical Societies as are recognized by representation in their respective State Societies, and from the Medical Department of the Army and Navy of the United States."

Also, strike out the fourth paragraph of same Article, and insert:

"Each State, County, and District Medical Society, entitled to representation, shall have the privilege of sending to the Association one delegate for every ten of its regular resident members, and one for every additional fraction of more than half that number.

"The Medical Staffs of the Army and Navy shall be entitled to four delegates each."

By Dr. P. PINEO, of Massachusetts:

Art. II., second paragraph, after "Army and Navy," insert "and the Marine Hospital Service of the United States."

By-Laws, Section 6, after "the chiefs of the bureaus of the Army and Navy," insert "and the Supervising Surgeon of the United States Marine Hospital Service."

By Dr. E. L. HOWARD, Maryland:

Art. IV. Strike out second clause of first paragraph, and insert:

"They shall be nominated by the Judicial Council, and shall be elected by vote on a general ticket."

By Dr. A. S. MAXWELL, of Iowa:

"*Resolved*, That in view of the many and important duties imposed upon the Nominating Committee, the Medical Society of each State and Territory that elects delegates be requested, when selecting delegates, to nominate one member of such delegation as their member of the nominating committee, and also designate the mode of filling vacancies."

By Dr. A. M. Pollock, of Pennsylvania:

Art. VI., first paragraph, strike out the word "five" and insert "ten."

By-Laws, Art. 5, first paragraph, strike out "five" and insert "ten."

We would call especial attention to the following from the Secretary:

Secretaries of all medical organizations that have adopted the Code of Ethics are respectfully requested to forward to the undersigned a complete list of their officers, with their post-office addresses, and the number of their members in good standing. This is the only guide for the Committee of Arrangements in determing as to the reception of delegates.

It will also enable the Permanent Secretary to present a correct report of the medical organizations in fellowship with the Association.

WM. B. ATKINSON, M. D.,
*Permanent Secretary.*

